# Supplementary material for: A GABA-receptor agonist reduces pneumonitis severity, viral load, and death rate in SARS-CoV-2-infected mice
Source: Front Immunol. 2022 Oct 25;13:1007955. doi: 10.3389/fimmu.2022.1007955 (PMC9640739; doi:10.3389/fimmu.2022.1007955)
Supplement: Supplementary file 1 [file DataSheet_1.pdf]

Supplementary Material

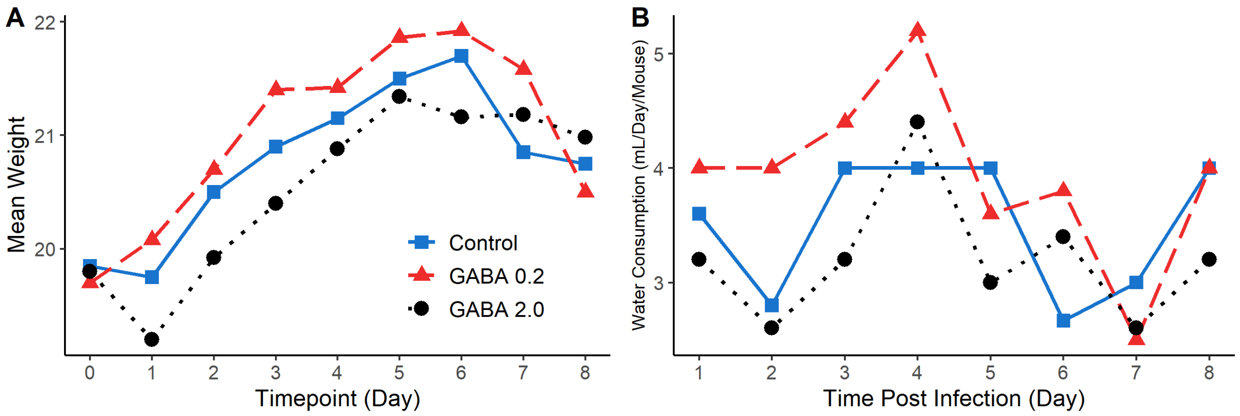

Supplementary Figure 1. (A) Longitudinal body weights of treatment groups following SARS-CoV-2 infection. Following SARS-CoV-2 infection, the mice were placed on plain water or water containing GABA at the indicated dose for the entire observation period. Their body weights were measured daily. Mice were euthanized when they had an illness score of 5 or at the end of the observation period (8 days post-infection). Data shown is mean body weight for surviving mice in each group. (**B) Average daily amount of water consumed.** We monitored how much water was consumed daily per cage and divided that by the number of surviving mice in the cage to calculate the average daily water consumption per mouse. For each treatment, we began with 5 mice/group with each group was housed in a single cage. Data shown is the average daily water consumption (mL) per mouse post-infection.
